# Early Functional Deficit and Microglial Disturbances in a Mouse Model of Amyotrophic Lateral Sclerosis

**DOI:** 10.1371/journal.pone.0036000

**Published:** 2012-04-25

**Authors:** Yannick Nicolas Gerber, Jean-Charles Sabourin, Miriam Rabano, Maria d M Vivanco, Florence Evelyne Perrin

**Affiliations:** 1 INSERM U1051, Institute for Neurosciences of Montpellier, Pathophysiology and Therapy of Sensory and Motor Deficits, Saint Elio Hospital, Montpellier, France; 2 IKERBASQUE Basque Foundation for Science, Bilbao, Spain; 3 Integrative Biology of Neurodegeneration, Neuroscience Department, University of the Basque Country, Leioa, Spain; 4 CIC bioGUNE, Cell Biology & Stem Cells Unit, Technological Park of Bizkaia, Derio, Spain; National Institute of Health, United States of America

## Abstract

**Background:**

Amyotrophic lateral sclerosis (ALS) is a neurodegenerative disorder characterized by selective motoneurons degeneration. There is today no clear-cut pathogenesis sequence nor any treatment. However growing evidences are in favor of the involvement, besides neurons, of several partners such as glia and muscles. To better characterize the time course of pathological events in an animal model that recapitulates human ALS symptoms, we investigated functional and cellular characteristics of hSOD1^G93A^ mice.

**Methods and Findings:**

We have evaluated locomotor function of hSOD1^G93A^ mice through dynamic walking patterns and spontaneous motor activity analysis. We detected early functional deficits that redefine symptoms onset at 60 days of age, i.e. 20 days earlier than previously described. Moreover, sequential combination of these approaches allows monitoring of motor activity up to disease end stage. To tentatively correlate early functional deficit with cellular alterations we have used flow cytometry and immunohistochemistry approaches to characterize neuromuscular junctions, astrocytes and microglia. We show that (1) decrease in neuromuscular junction's number correlates with motor impairment, (2) astrocytes number is not altered at pre- and early-symptomatic ages but intraspinal repartition is modified at symptoms onset, and (3) microglia modifications precede disease onset. At pre-symptomatic age, we show a decrease in microglia number whereas at onset of the disease two distinct microglia sub-populations emerge.

**Conclusions:**

In conclusion, precise motor analysis updates the onset of the disease in hSOD1^G93A^ mice and allows locomotor monitoring until the end stage of the disease. Early functional deficits coincide with alterations of neuromuscular junctions. Importantly, we identify different sets of changes in microglia before disease onset as well as at early-symptomatic stage. This finding not only brings a new sequence of cellular events in the natural history of the disease, but it may also provide clues in the search for biomarkers of the disease, and potential therapeutic targets.

## Introduction

Amyotrophic lateral sclerosis (ALS) is a chronic neurodegenerative disease characterized by a selective degeneration of motoneurons in the motor cortex, brainstem, and spinal cord. Symptoms start with progressive weakness, atrophy of skeletal muscles and paralysis that eventually lead to death. The prevalence of ALS is of 2–5 per 100 000 individuals and the median survival of 36 months after symptom onset. Approximately 90% of ALS patients are sporadic cases with no known genetic component whereas 10% are familial cases. In familial ALS 20% of the patients harbour a mutation in the gene coding for the enzyme Cu/Zn superoxide dismutase 1 (SOD1) (for reviews see [1], [2]).

Transgenic mice over-expressing the human mutated gene for Cu/Zn SOD1 had been created based on the identification of missense mutations in the human SOD1 gene responsible for one form of familial ALS [3]. The most extensively used SOD1^G93A^ transgenic line harbours a mutant form of human SOD1 containing a Gly^93^→Ala amino acid substitution [4]. These mice develop a dominantly inherited adult-onset paralytic disorder that recapitulates human ALS symptoms. Disease onset is described at around 80–90 days of age with progressive clinical weakness followed by paralysis and death by 135 days.

Pathogenesis of motoneuron degeneration in ALS and mechanisms of selective vulnerability are still largely unknown although it has been demonstrated that ALS is a complex multifactorial disease (protein misfolding, glutamate-mediated excitotoxicity, oxidative stress, impaired axonal transport) that involves several cellular partners such as neuron, glial and muscle cells (for reviews see [1], [2], [5], [6]). Thus, there is growing interest about whether the death phenomenon in motoneurons is indeed cell autonomous or mediated by non-neuronal cells [7], [8], [9], [10], [11]. A better understanding of the role of these neighboring cells has resulted in the recognition of the important role played by astrocytes and microglia in health and disease (for review see [5]). Moreover, the presence of reactive astrocytes and microglia is a hallmark of ALS [12], [13].

Studies on human cells and tissues - both normal and diseased - are obviously critical for moving forward with any particular therapeutic strategy, but these types of studies are limited due to their complexity and to the limited access to biopsy material. For this reason, it appears mandatory to better characterize the time course of pathological events in animal models of the disease.

In this study, through dynamic walking patterns analysis of hSOD1^G93A^ transgenic mice we have revised motor symptoms onset at 60 days of age, i.e. 20 days earlier than previously described. Moreover, sequential combination with spontaneous motor activity analysis has allowed locomotor activity monitoring up to the end stage of the disease.

In parallel, tissular and cellular studies have demonstrated that early functional deficits coincide with alterations of neuromuscular junctions. Finally, we have identified a very early cellular hallmark of the disease in microglia, which have never been before considered as key elements in the early pathogenesis of this disease.

## Results

### Locomotor function is altered at 60 days of age in hSOD1^G93A^ mice

To better characterize disease onset and progression in the hSOD1^G93A^ mice we have associated two behavioral tests that allow the evaluation of locomotor function.

**Figure 1:**
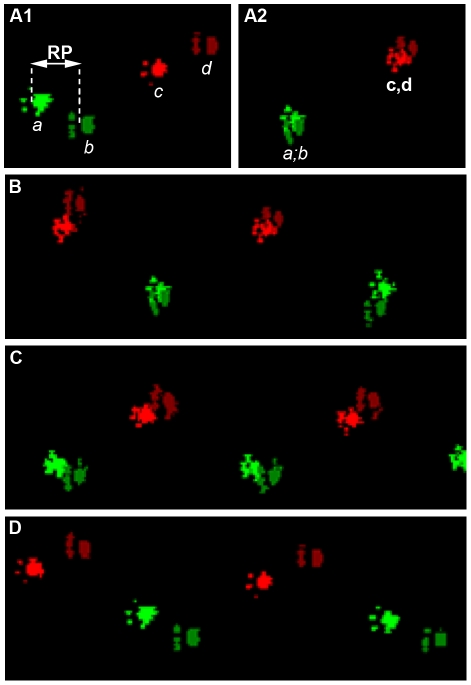
CatWalk™ patterns of hSOD1^G93A^ mice. A1 – The “relative position" corresponds to the distance between the placement of front limb and hind limb paws over one walking step. (A1 a–c) - Front paws are represented by glossy colour and (A1 b–d) hind paws are represented in matt. A1 - Example of a full step cycle in hSOD^G93A^ mice at 90 days of age and A2 - control littermates. B - Walking pattern of control mice, C - transgenic animals at 60 days of age and D - 90 days of age. a: right front limb paw, b: right hind limb paw, c: left front limb paw, d: left hind limb paw.

**Figure 2:**
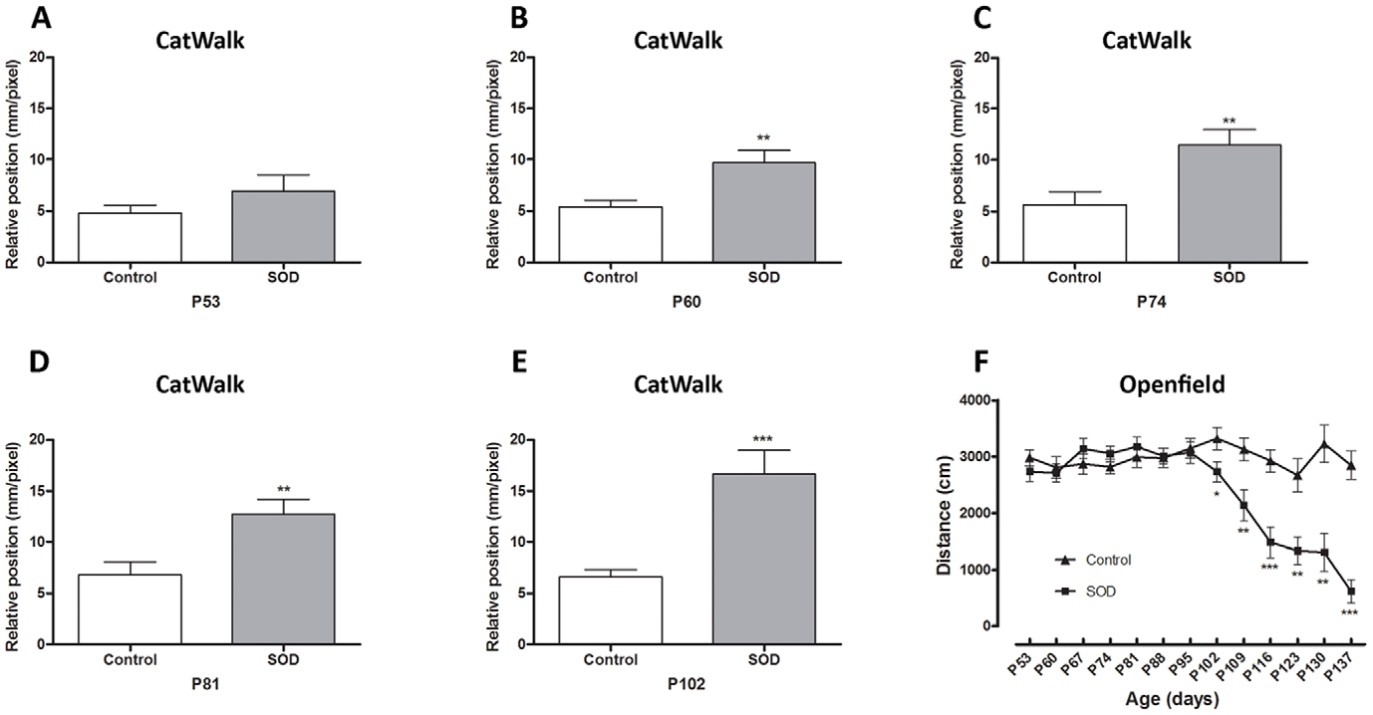
Locomotor function analysis of hSOD1^G93A^ mice. (A–E) - CatWalk™ gait analysis. Graphs represent the distance between the front and hind paws on a step sequence (“relative position"; see Figure 1 for details) of control and hSOD1^G93A^ animals. A, B, C, D and E - Respectively 53, 60, 74, 81 and, 102 days of age. F - Open field test. Spontaneous locomotor activity represented by the mean distance covered by control and hSOD1^G93A^ mice in a 8 minutes recording test over disease progression. For CatWalk™ analysis a minimum of 7 animals and up to 13 per group and time point were included (see selection criteria in Supplementary Figure 1). For Open field test a minimum of 20 animals were used from day 53 to 116, then due to the death of hSOD1^G93A^ mice, a minimum of 5 animals were analyzed (P123 to P137). Statistics: t-test; * p<0.05, ** p<0.01 and *** p<0.001.

We have used the CatWalk™ system to carry out an automated dynamic gait analysis and the open field approach to record spontaneous motor activity. Mice were tested on a weekly basis from 50 days of age. Open field analysis was done until the end stage of the disease whereas CatWalk™ testing had been carried out up to 105 days of age since then mice were too paralyzed to achieve the test. The CatWalk™ system yields quantitative gait parameters, including the “relative position" of the paws that correspond to the distance between front and hind footprints over a step cycle (***Figure 1 A1&A2***). In a step cycle, control animals place their hind paws in the footprint of the front paws (***Figure 1B***, glossy and matt prints correspond respectively to front and hind paws). This gait pattern is a very robust parameter in control littermates. At 53 days of age hSOD1^G93A^ animals presented the same pattern as controls (***Figure 2A***), whereas from 60 days onwards, they showed a subtle but constant alteration in gait pattern appearing as the inability to draw their hind limbs up to the previous position of their front limbs (***Figure 1C*** and ***Figure 2B***). This alteration in gait pattern increased further up to 102 days of age (***Figure 1D*** and ***Figure 2C–E***). Using the CatWalk™ system, we thus demonstrate that locomotor alteration in the hSOD1^G93A^ mouse model starts 20 days earlier than described in the literature. This previous disease onset corresponds in our experiments to the significant decrease in weight of the mutated animals as compared to controls observed from 84 days of age (***Figure S1***).

We used the open field system to measure the spontaneous motor activity of hSOD1^G93A^ and control mice. We highlighted a decreased in the mean distance covered by transgenic animals as compared to their control littermate from 102 days of age until the end stage of the disease (***Figure 2F***).

Taken together, these results demonstrate that the combined use of CatWalk™ and open field analysis systems allows a thorough description of motor alterations in the hSOD1^G93A^ mouse model. CatWalk™ analysis allowed early detection of motor symptoms and open field permitted a follow up of motor activity up to the end stage of the disease.

### Decrease in number of neuromuscular junctions correlates with motor impairment

**Figure 3:**
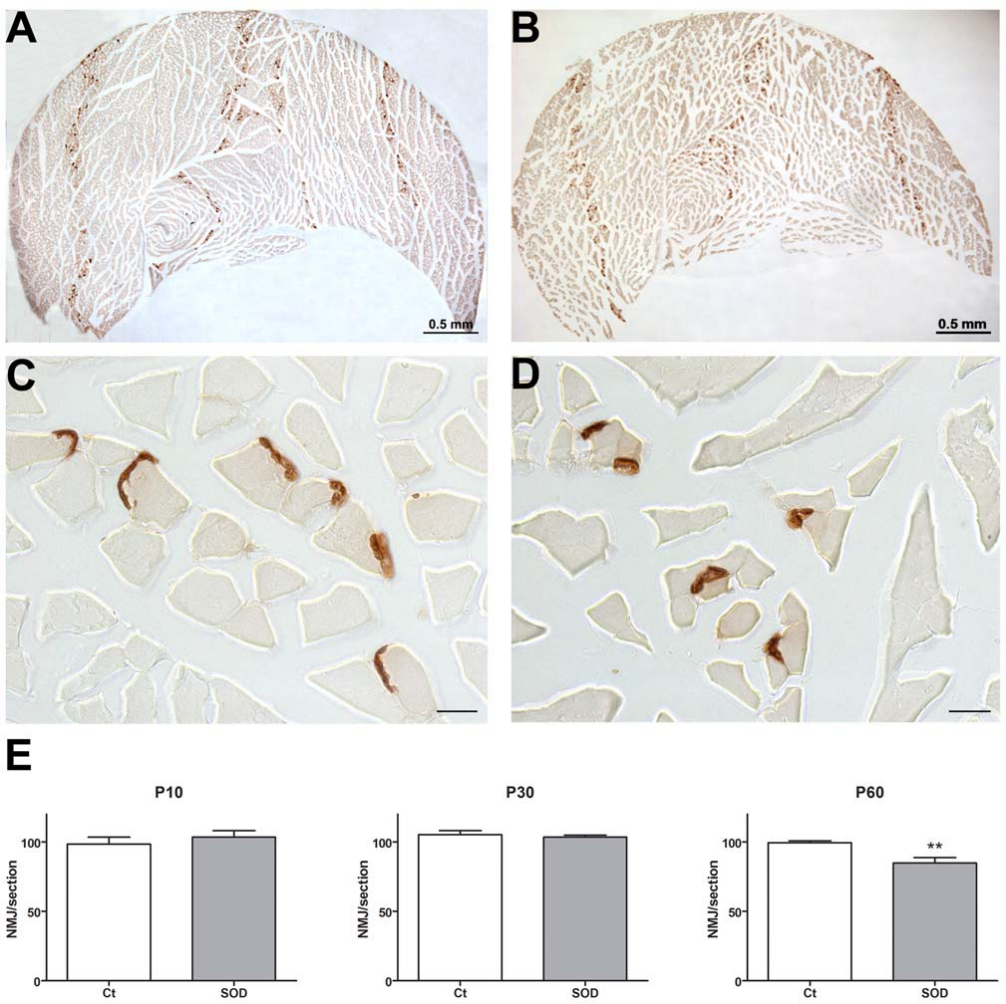
Neuromuscular junctions in the gastrocnemius-soleus-plantaris complex in control and hSOD1^G93A^ mice at pre-and early-symptomatic ages. Neuromuscular junctions were revealed by the Karnovsky and Roots enzymatic method. **A** - Low magnification photograph of the *gastrocnemius-soleus-plantaris* complex from a P30 control mouse. B - Low magnification photograph of the *gastrocnemius-soleus-plantaris* complex from a P30 hSOD1^G93A^ mouse. **C** - High magnification photograph of the *gastrocnemius* from a P60 control mouse. **D** - High magnification photograph of the *gastrocnemius* from a P60 transgenic mouse. **E**- Quantification of the neuromuscular junctions at two pre-symptomatic ages (P10 and P30) and at early-symptomatic age (P60). Statistics: t-test; ** p<0.01.

Such alterations of the locomotor pattern suggest an early impairment of skeletal hind limbs muscles. This prompted us to examine neuromuscular junctions in the *gastrocnemius-soleus-plantaris* complex at symptoms onset (P60) (***Figure 3C–E***). Using an enzymatic method that reveals cholinesterases we confirmed a reduction in neuromuscular junction's number in hSOD1^G93A^ animals as compared to control littermates (99.38±1.399 vs. 84.71±3.931, p = 0.0079). At the two pre-symptomatic ages we examined, (P10 and P30, ***Figure 3A, B&E***) neuromuscular junction's number was not different in control and transgenic animals. These results demonstrate morpho-functional correlation in the early onset of symptoms affecting hind limbs in hSOD1^G93A^ mice.

### Astrocytes number is not altered at pre-and early-symptomatic ages but spinal repartition is modified at symptoms onset

**Figure 4:**
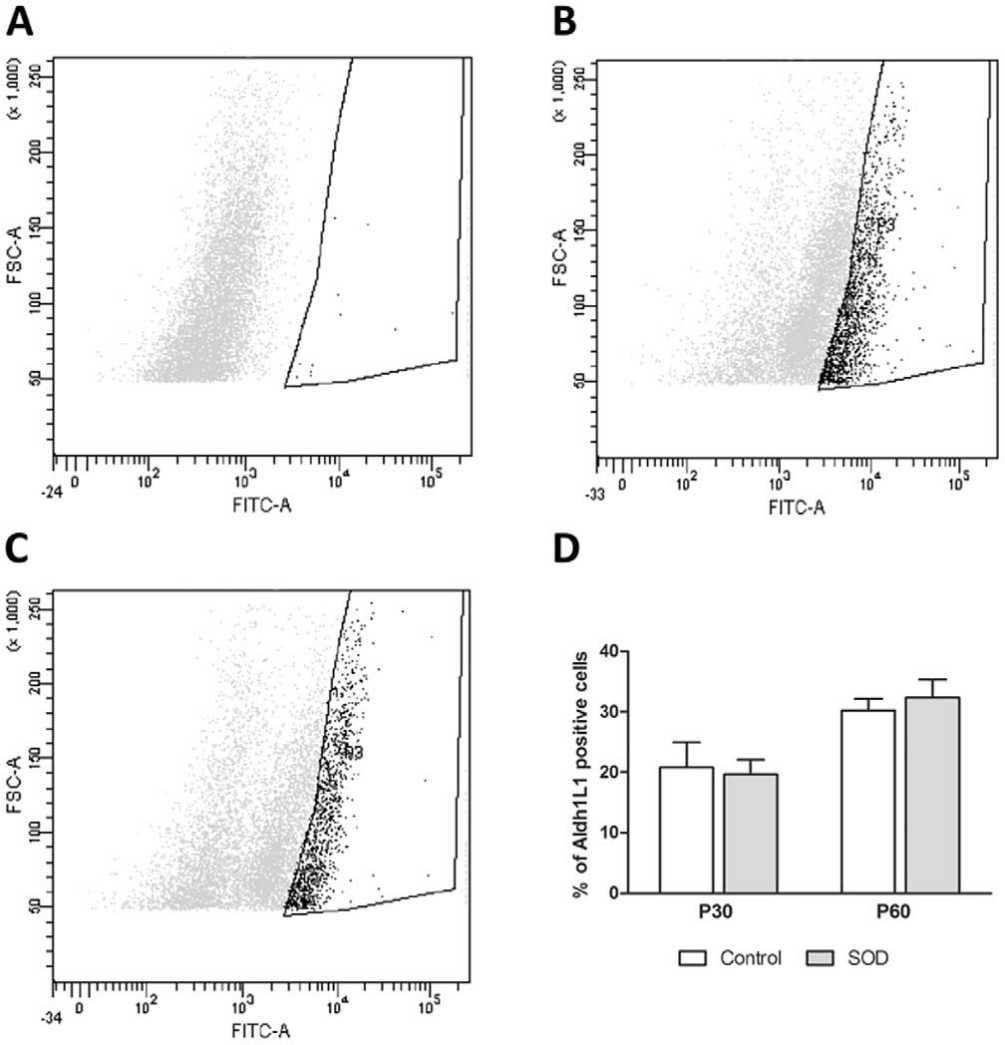
FACS analysis of astrocytes in control and hSOD1^G93A^ mice spinal cords. The number of spinal astrocytes from hSOD1^G93A^ animals and their control littermates was assessed by flow cytometry using the pan-astrocyte marker Aldh1L1. (A–C) - Representative flow cytometry analysis dot plot astrocyte profiles. A - Negative control (without Aldh1L1 labeling). B - Control and C - hSOD1^G93A^ spinal astrocytes at P60. In both B -controls and C hSOD1^G93A^ surrounded areas, designed as “P3", correspond to the labeled cells. D - Quantification of Aldh1L1^+^-astrocytes in control and hSOD1^G93A^ animals at pre-symptomatic (P30) and early-symptomatic (P60) stages. The X-axis represents the intensity of fluorescence and the Y-axis the size of the cells.

There is growing evidences of the involvement of glial cells in ALS. We thus investigated possible astrocyte modifications at pre-symptomatic and early symptomatic ages. To evaluate the total number of astrocytes in the spinal cord of hSOD1^G93A^ and control animals, we used flow cytometry analysis based on the pan-astrocytic marker Aldh1L1 to obtain the widest astrocytic population. We did not observe any difference in the number of Aldh1L1^+^ -astrocytes in hSOD1^G93A^ as compared to the controls at both pre-symptomatic (P30; 16.52±4.899 n = 3 in the control group vs. 19.70±2.364 n = 3 in transgenic animals) and symptomatic ages (P60; 22.91±4.954 n = 4 in the controls vs. 23.83±4.717 n = 4 in transgenic animals) ***(*** ***Figure 4*** ***and Figure S2)***.

Astrogliosis is a hallmark of ALS end-stage. In order to better quantify possible gliosis, we have used the classical astrocytic marker GFAP. We first validated the accuracy of our FACS approach by comparing transgenic and control animals at end-stage of the disease (P120). As expected, spinal cords of transgenic animals exhibit a 1.6-fold increase in the number of GFAP^+^-astrocytes ***(Figure S3I–L)***. We thus performed FACS analyses for GFAP at pre-symptomatic (P30) and early-symptomatic (P60) stages. This analysis did not show any significant difference between transgenic and control animals ***(Figure S3A–H)***.

**Figure 5:**
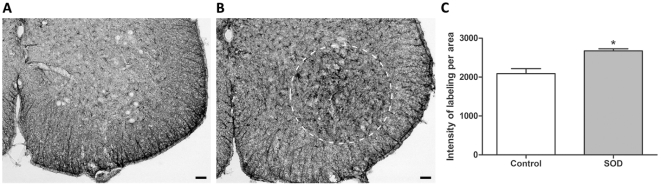
Spinal cord astrocytes in control and hSOD1^G93A^ mice at early-symptomatic stage. (A–B) - Photographs of GFAP immunohistochemical labeling from A - control or B - hSOD1^G93A^ lumbar spinal cord section at early-symptomatic (P60) stage. Dotted circle in (B) delineate astrogliosis. (C) Quantification of the mean labeling intensity per area in the lumbar spinal cord of control or hSOD1^G93A^ animals. Statistics: t-test; * p<0.05. Scale bars (A–B): 50 µm.

However at early symptomatic stage, we highlighted using immunohistochemistry, differences in astrocytes repartition. Lumbar spinal cords from transgenic animals indeed displayed astrogliosis in the spinal ventral gray matter as compared to the controls ***(*** ***Figure 5A–B*** ***)***. Quantification of the mean labeling intensity confirmed this observation since transgenic animals presented a 1.3-fold increase of GFAP in the ventral gray matter as compared to controls (2674±52.44 n = 3 vs. 2088±129.1 n = 3) ***(*** ***Figure 5C*** ***)***.

These results demonstrate, using two specific markers, that there is no difference in astrocyte number in the entire spinal cord between control and hSOD1^G93A^ mice before disease onset and at early-stage of the disease. Nevertheless, astrogliosis is already visible in the lumbar spinal ventral horn of transgenic animals at early-symptomatic stage.

### Microglia modifications precede disease onset

**Figure 6:**
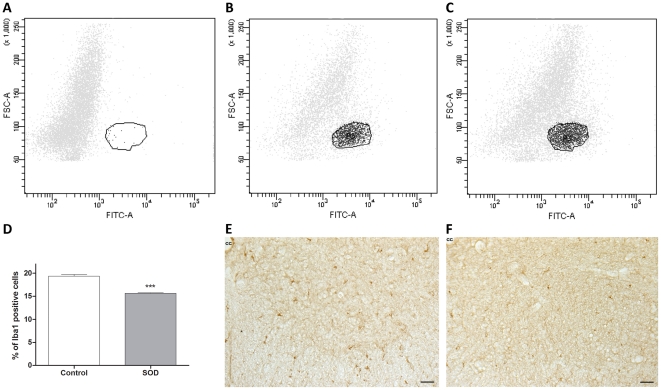
Spinal cord microglia in control and hSOD1^G93A^ mice at a pre-symptomatic stage. The number of microglia from hSOD1^G93A^ animals and their control littermates was assessed by flow cytometry using the microglial marker Iba1. (A–C) - Representative flow cytometry analysis dot plot microglia profiles. A - Negative control (without Iba1 staining). B - Control and C - hSOD1^G93A^ spinal microglia at P30. In both B - controls and C - hSOD1^G93A^ surrounded areas, designed as “P3", correspond to the labeled cells. D - Quantification of Iba1^+^-microglia in control and hSOD1^G93A^ animals at pre-symptomatic (P30) age. (E–F) - Photographs of Iba1 immunohistochemical labeling from E - a control or F - hSOD1^G93A^ lumbar spinal cord section. The X-axis represents the intensity of fluorescence and the Y-axis the size of the cells. Statistics: t-test; *** p<0.001. Scale bars 50 µm.

A second glial cell type that may be involved in ALS is microglia. We have used the same FACS approach to quantify the number of microglia in the spinal cord of hSOD1^G93A^ and control animals at pre- and early-symptomatic stages using the microglial marker Iba1. Surprisingly, we have found a decrease in microglia percentage (vs total number of cells) in the entire spinal cord at the pre-symptomatic age (P30) in transgenic animals compared to their control littermate (19.37±0.362 n = 3 for the control vs. 15.66±0.094 n = 3 for the transgenic; p = 0.0006) ***(*** ***Figure 6A–D*** ***)***. In order to determine microglia repartition and morphology, we performed immunolabeling for Iba1 on lumbar spinal cord sections from control and transgenic animals. We did not notice any difference in microglial morphology. ***(*** ***Figure 6E–F*** ***)***.

**Figure 7:**
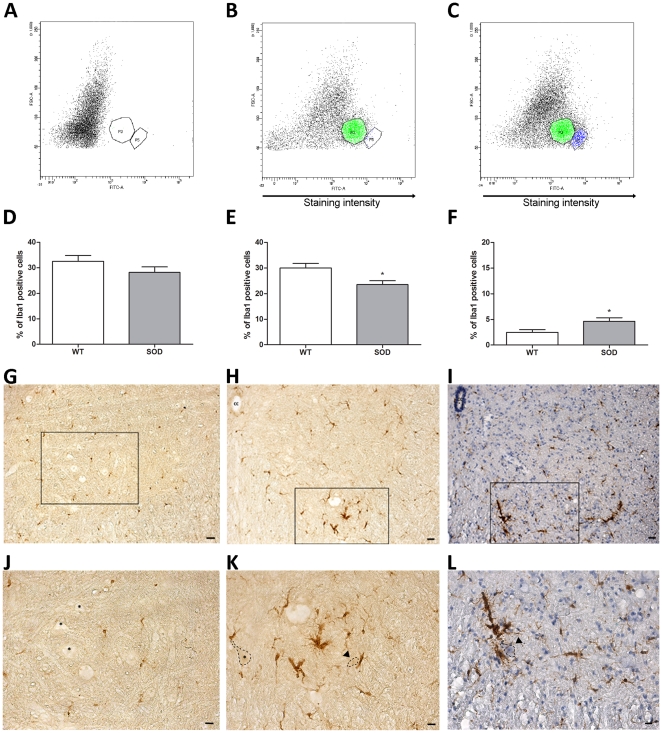
Spinal cord microglia in control and hSOD1^G93A^ mice at an early-symptomatic stage. The number of microglia from hSOD1^G93A^ animals and their control littermates was assessed by flow cytometry using the microglial marker Iba1. The abscissa represents the staining intensity. (A–C) - Representative flow cytometry analysis dot plot microglia profiles. A - Negative control (without Iba1 staining). B - Control and C - hSOD1^G93A^ spinal microglia at P60. In both B - Controls and C - hSOD1^G93A^ surrounded areas, designed as “P3", correspond to the Iba1^low^ cells (same microglia population as in P30 experiment). Areas designed as “P5" correspond to the Iba1^high^ cells. (D–F) - Quantifications of microglia in spinal cord of Control and hSOD1^G93A^ animals at early-symptomatic (P60) age. D - Total Iba1^+^-microglia, E - Iba1^low^ microglia and F - Iba1^high^ microglia. (G–I) - Photographs of Iba1 immunohistochemical labeling from G - control or H - hSOD1^G93A^ lumbar spinal cord section. I - Combination of Iba1 immunolabeling and hematoxylin on a hSOD1^G93A^ lumbar spinal cord section. (J–L) - High magnifications of the boxes in (G, H and I). Asterisks correspond to healthy motoneurons. Arrowheads show degenerating motoneurons surrounding by activated microglia. The X-axis represents the intensity of fluorescence and the Y-axis the size of the cells. Statistics: t-test; * p<0.05. Scale bars (G–I): 40 µm; (J–L): 20 µm.

At early-symptomatic age (P60), we did not see any difference in the total number of Iba1^+^-microglia in the entire spinal cord (32.53±2.276 n = 5 in controls vs. 28.22±2.167 n = 3 in hSOD1^G93A^) ***(*** ***Figure 7D*** ***)***. However, FACS analysis on transgenic animals separated two distinct microglia sub-populations ***(*** ***Figure 7A–C*** ***)***: one with a low level of Iba1 expression (Iba1^low^ corresponding to “P3" area) and one with a high level of Iba1 expression (Iba1^high^ corresponding to “P5" area). Although the total number of microglia was similar in both control and transgenic animals, we found that percentages of the two microglia sub-populations were different. Indeed, Iba1^low^ microglia is significantly decreased in transgenic animals whereas the Iba1^high^ population is increased about 2-fold in transgenic as compared to their control littermate (respectively: Iba1^low^: 30.05±1.780 n = 5 in controls vs. 23.56±1.487 n = 3 in transgenics; p = 0.0474. Iba1^high^: 2.480±0.523 n = 5 in controls vs. 4.657±0.688 n = 3 in transgenics; p = 0.0445) ***(*** ***Figure 7D–F*** ***)***. This difference could reflect a variation in activation states. In order to further substantiate this point, we have examined Iba1 expression on lumbar spinal cord sections from control and hSOD1^G93A^ animals. We observed a clear microglial activation in the ventral spinal horn of transgenic animals as compared to control. Indeed, microglia from control animals displayed a small soma associated with fine ramified processes that are characteristics of resting microglia ***(*** ***Figures 7G and J*** ***)*** whereas transgenic animals harbored microglia with large soma and reduced processes complexity ***(*** ***Figures 7H and K*** ***)***. Moreover, the ventral lumbar spinal horn of transgenic animals presented microglial density characteristic of inflammatory state (***Figures 7I and L*** ***)***. Interestingly, activated microglia were mainly located in close vicinity of morphologically altered motoneurons (reduced size and condensated nuclei) (arrowheads in ***Figure 7K–L***).

Thus, different sets of changes in microglia before disease onset, on the one hand, as well as at early-symptomatic stage, on the other hand, are in favor of their potential involvement in the early phases of the disease.

## Discussion

### Generalities

Our findings on the locomotor function of hSOD1^G93A^ mice indicate that deficits are detectable from 60 days of age, this updating functional symptoms of disease onset when compared with the previously admitted 80–90 days of age. Moreover we show that a sequential combination of two behavioural tests allows the accurate monitoring of motor alteration from disease onset until its end stage. When looking at the periphery, we found that early functional motor deficits correlate with a decrease in motor end-plates number. Finally, in the spinal cord of transgenic animals, we discovered a pre-symptomatic decrease in microglia number, whereas astrocytes are not affected. At early symptomatic age we describe a complex alteration of astrocyte and microglia organization in hSOD1^G93A^ mice.

### Locomotor function is altered at 60 days of age in hSOD1^G93A^ mice

Transgenic mice on a B6SJL background carrying a high copy number of the G93A human SOD1 mutation (B6SJL-Tg (SOD1-G93A) 1Gur/J) is the most commonly used mouse model of ALS. The majority of pre-clinical studies and clinical trials are based on results obtained with this transgenic strain. So far, translation of the results obtained in mice to human has been disappointing. This may be due to an inaccurate characterization of the animal model, and in particular of the lack of sensitivity of the behavioural analysis that had been used and may not allow the complete and precise symptoms follow up in transgenic mice.

Using an automated gait analysis system, we report early functional deficits that redefine symptoms onset at 60 days of age, i.e. 20 days earlier than previously described. Our results confirm and extend recent data obtained with the digigait treadmill video based system on the same transgenic strain [14]. However, noteworthy is that the CatWalk™, through the dynamic analysis of walking patterns, and conversely to the treadmill, does not induce any physical exercise that could affect the lifespan of the mice [15], [16], [17]. Moreover, the reproducibility of the digigait analysis system is still under debate [18], [19]. The CatWalk™ system seems thus better adapted for the evaluation of therapeutic approaches. In a recent study, early motor symptoms had been detected at 45 days of age using a rotarod in SOD1^G93A^ mice on a C57/BL/6 background. In the same study modifications of step patterns were identified around P75 using CatWalk™ system. One of the drawbacks of the use of the rotarod in the assessment of a therapeutic approach is its influence on the pathophysiology of the disease, such as increases of the lifespan and cell proliferation that have been reported in a mouse model of motoneuron degeneration [15]. Discrepancy in CatWalk™ results obtained in this study and our data could be due to the difference in genetic background. Moreover, and conversely to the previous study, we detect significant and reproducible motor deficits in paw placement from day 60 to 102. We can thus conclude that using the CatWalk™ analysis resets early locomotor functional deficits at 60 days of age in hSOD1^G93A^ mice and reinforces the accuracy of the automated gait analysis method in neurodegenerative diseases [20]. In the gastrocnemius muscular complex, we show that the decrease in the number of neuromuscular junctions at 60 days of age correlates with the early symptom onset. Interestingly, we correlate this functional deficit with the concomitant decrease in the number of motor end plates in the gastrocnemius muscular complex. These data confirm previous results obtained [21], [22] with other enzymatic based method [23].

Moreover, we provide a complete set of evaluation of the spontaneous locomotor activity from day 60 to the end stage of the disease, through the association of the CatWalk™ with the open field. This is of importance in the perspective of translational studies. Indeed, key issues to assess the potential effects of a given therapeutic approach are a precise characterization of the symptomatic period, and the accurate monitoring of the progression of the paralysis in this mouse model. This approach can be extended to other neuromuscular disorders.

### Glial alterations at pre-and early-symptomatic ages

**Astrocytes:** Astrogliosis is a hallmark of amyotrophic lateral sclerosis in patients where reactive astrocytes surround both upper and lower motor neurons [13]. In the spinal cord of ALS patients, GFAP-immunoreactivity is particularly noticeable in the grey matter of the ventral horn where normally astrocytes express GFAP at very low levels [24].

A similar pattern of astrogliosis has been described in many transgenic ALS mouse models, although the time course of astrogliosis, relative to motor neuron degeneration and symptoms onset, seems to vary depending on the model. However, astrocyte reactivity generally precedes motoneuron degeneration and symptoms onset. In hSOD1^G37R^ mice, astrogliosis is indeed described as early as 5 weeks of age whereas motoneurons death typically occurs around 15 weeks [25]. In the hSOD1^G85R^ mouse model, Bruijn et al. have shown SOD1 and ubiquitin astrocytic inclusions as initial indicators of the disease and presence of reactive astrocyte becomes evident at 6.5 months of age i.e. 1 month before motoneuron loss [26].

Using a combination of FACS analysis and immunohistochemistry, we have investigated spinal astrocyte population in hSOD1^G93A^ mice at the pre- and early-symptomatic stages (respectively P30 and P60) as determined previously. FACS analysis did not reveal any difference in astrocyte number either with the pan-astrocytic marker Aldh1L1 or with GFAP. However, at the early-symptomatic stage, histological examination detected an increase in GFAP labelling in the ventral horn of lumbar spinal cord of transgenic animals as compared to controls. This is thus the first description of astrogliosis occurring before motor neuron loss in the hSOD1^G93A^. Indeed, astrocyte reactivity had been previously described at 90–100 days of age [27], [28] whereas motoneuron degeneration has been observed as early as 74 days of age [29].

**Microglia:** There are growing evidences for the involvement of microglia, the resident macrophage of the CNS, and an immune response in ALS (for reviews see [30], [31], [32]). Indeed activated microglia and T cells are one of the components of ALS (for review see [32]). Microglia, that survey the neural environment for potential intrusion, are in constant molecular interaction with other cell types and are characterized by an early activation in response to injury. In a physiological context, microglia synthesize and release trophic factors whereas in a pathological circumstances, they rapidly switch from a resting to an activated state. Activation results in morphological changes from ameboid to phagocytic cells with numerous processes and proliferation. Moreover, in a pathological context, microglia most likely play a dual detrimental and beneficial effect. On the one hand, through the release of neurotrophic factors and anti-inflammatory cytokines they are hypothesized to exert a neuroprotective action and on the other hand, the release of pro-inflammatory cytokines and free radicals may be cytotoxic for neurons (for review see [31]).

In ALS animal models microglia activation has been reported at the early stage of the disease and increased with disease progression (for review see [31]). In particular, in hSOD1^G93A^ mice microglia activation in the ventral spinal horn is evident before the previously accepted date of clinical onset (i.e. 80 days of age) [12], [27]. It has been hypothetized that in this animal model, the dialogue between motoneurons and microglia initially protects motoneurons (for review see [30]).

Microglial activation, revealed by CD11b labelling, had been reported at 32 days of age in the lumbar ventral horn of hSOD1^G93A^ mice [33]. In our study, we describe for the first time a deficit in microglia number in the entire spinal cord of hSOD1^G93A^ mice at very early age, i.e. 30 days of age. This result is not in opposition with the previous study of Saxena et al. [33] since at variance with them, we have not restricted our analysis to the lumbar part of the spinal cord and we did not use the same microglia markers. The deficit in microglia number that we observed may reflect an intrinsic alteration in this specific cell population and argues for of an immune deficit. A reduction in number, proliferation and maturation of T cells has been recently reported in hSOD1^G93A^ mice and ALS patients [34], [35]; and T cells, acting by modulation of microglia inflammatory response have been suggested to play a neuroprotective role (for review see [36]).

At 60 days of age, that correspond to early symptomatic stage, we do not find a difference in total microglia number but we identify an increase in a subpopulation of microglia that express high levels of Iba1. This increase in Iba1 expression is a sign of an activation of microglia [37], [38], we indeed found with immunohistochemistry a morphology representative of activated microglia and substantiate a previous study in hSOD1^G93A^ rats [39]. This most likely reflects an early phase of proliferation since in ALS animal models, activated microglia arise mainly from endogenous origin rather than resulting from peripheral recruitment [40], [41], thus increase in number most likely reflects local microglia proliferation [32].

Our examination of glial cells, that evidences a complex sequence of alterations in astrocytes and microglia populations before or concomitantly to motoneuron degeneration, strongly suggests an early implication of glia in the initiation of the disease. It may also provide clues in the search for biomarkers of the disease, and for potential therapeutic targets.

In summary, the present study has brought forward a hitherto unrecognized set of functional and anatomical hallmarks of the hSOD1^G93A^ mouse model of ALS, thus providing new clues for translational studies of this devastating disease.

## Methods

### Animals

Experimental procedures followed the European legislative, administrative and statutory measures for animal experimentation (86/609/EEC) and the Declaration of Helsinki. The study was approved by the “Direction des Services Vétérinaires de l'Hérault", France (authorization number 34118) and ratified by the “Préfecture de l'Hérault", France. Every effort was made to minimize the number of animals and their suffering. Transgenic mice carrying the G93A human SOD1 mutation B6SJL-Tg (SOD1-G93A) 1Gur/J (ALS mice) were purchased from The Jackson Laboratory (Bar Harbor, ME, USA) and bred on a B6SJL background. Transgenic mice were identified by PCR and housed in controlled conditions (hygrometry, temperature and 12 h light/dark cycle). Only males were used and litter-matching between groups were done as much as possible. Record of the weight was done once a week from 56 days onward.

### Behavioural analysis

**CatWalk™:** The CatWalk™ is a video-based automated gait analysis system that allows to determine dynamic and voluntarily walking patterns in rodent models (Noldus, Wageningen, The Netherlands). The principle is based on an optical technique. In a dark room, a glass walkway floor is enlightened by a fluorescent tube. When the animal crosses the walkway, the light is refracted by any contact on the glass. Different paw contacts and placements are visualized and recorded by a camera. The CatWalk™ system measures various aspects of locomotor pattern. Indeed, based on the position, pressure, and surface area of each footfall, multiple parameters are calculated, amongst those, the “relative position" corresponds to the distance between the placement of front and hind paws over one walking step (***Figure 1A1***).

For data collection, six runs per animal were performed on a weekly basis from day 53 and until animals were not able to do the test. For each mouse a minimum of three runs crossed at the same speed with 3-full step sequence patterns per run were recorded.

To avoid bias due to stress, we placed transgenic and control littermate mice on the glass plate 7 and 3 days prior to the first recording session. Recording sessions were stopped when the transgenic mice were not able to correctly cross the walkway due to hind limb paralysis.

**Open field activity:** Spontaneous locomotor activity of control and transgenic mice was monitored in an open field test. Animals were placed in an empty test arena (45×45 cm box) and movements automatically recorded by infra-red capture. We analyzed the total distance (cm) using the Actitrack soft system (Bioseb, Open field, Actitrack software, Vitrolles, France). Analysis started at P53 and until the end of life of the transgenic mice; analysis correspond to a weekly 8 minutes session preceded by 2 minutes without recording to avoid any bias due to stress.

### Immunohistochemistry

Mice were anesthetized with pentobarbital (i.p), and perfused intracardially with cold PBS followed by cold 4% paraformaldehyde (Sigma Aldrich, Saint Louis, USA). Spinal cords and *gastrocnemius - soleus - plantaris* muscular complexes were removed and post fixed for 24 h in 4% paraformaldehyde. Samples were cryoprotected in sucrose 30%, included in Tissue Teck (Sakura, Alphen aan den Rijn, Pays Bas), frozen and kept at −80°C until processing.

Free floating spinal cord transversal sections (20 µm) were washed twice in PBS (5 min), treated for 30 min in PBS containing lysine (20 mM, pH 7.4) and for 15 min in 1% H_2_O_2_). Sections were then permeabilized and blocked for 1 hour with PBS containing bovine serum albumin (BSA, 10%) (all from Sigma Aldrich, Saint Louis, USA) and Triton X-100 (0.1%) (Fisher Scientific, Illkirch, France) and then incubated 48 hours at 4°C with either Iba1 (1/1000; Wako Pure Chemical Industries, Osaka, Japan) or GFAP (1/2000; Dako, Glostrup, Denmark) primary antibodies diluted in the same solution.

Secondary rabbit peroxydase-conjugated antibody was used (1∶500; Jackson Immunoresearch, Carlsbad, USA). Sections were then washed in TRIS buffer and enzymatic and revelation was done with diaminobenzidine and H_2_O_2_ 0.1% as a substrate.

Some sections were counterstained with Mayer hematoxylin solution 0.1% for 15 minutes (Sigma Aldrich, Saint Louis, USA).

Quantifications of the mean labelling intensity were separately and blindly done by two experimenters using the Adobe® Photoshop® software (Adobe, San Jose, USA). One section out of twenty was used for quantification all along the lumbar spinal cord.

### Neuromuscular junctions labelling

For neuromuscular junctions (NMJ) labelling we followed the protocol of Karnovsky and Roots [23]. We analyzed the entire *gastrocnemius - soleus - plantaris* muscular complex on transverse sections (16 µm). NMJ Quantification was done blindly every 3 sections.

### FACS

hSOD1^G93A^ mice and control littermates were anesthetized with pentobarbital (2.5 ml/kg) and intracardially perfused with cold PBS to chase the blood. Spinal cords were dissected and then dissociated in 750 µl PBS (Invitrogen, Carlsbad, USA), 100 µl trypsin 13 mg/ml, 100 µl hyaluronidase 7 mg/ml, 50 µl kinurenic acid 4 mg/ml (all from Sigma Aldrich, Saint Louis, USA), and 20 µl DNAseI 10 mg/ml (Roche, Rotkreuz, Switzerland) for 30 minutes at 37°C. Final dissociation was made by pipetting. Cell suspension was sieved on a 40 µm sieve (BD Biosciences, Franklin Lakes, USA). To eliminate myelin, cells were re-suspended in PBS-0.9 M sucrose and centrifuged for 20 minutes at 750 g. The pellet was then fixed in cold 4% paraformaldehyde for 20 minutes.

For labeling, cells were incubated 10 minutes on ice in PBS-0.5% BSA-0.1% saponin (Sigma Aldrich, Saint Louis, USA). Primary antibodies were diluted in the same solution at the following dilutions: Iba1 1/500 (Wako Pure Chemical Industries, Osaka, Japan), GFAP 1/1000 (Dako, Glostrup, Denmark), Aldh1L1 1/1000 (Abcam, Cambridge, UK). After a 20 minutes incubation, cells were washed with cold PBS and re-suspended into the secondary antibody solution (donkey anti rabbit Alexa 488 1/1000 in PBS-0.5% BSA, (Invitrogen, Carlsbad, USA)) for 15 minutes on ice. Aldh1L1 labeling requires a demasking step in sodium citrate 10 mM pH6 for 2 hrs between fixation and blocking steps.

Cells were analyzed with a FACS ARIA (BD Biosciences, Franklin Lakes, USA), equipped with a 488 nm Laser Sapphire 488-20. Size threshold was used to eliminate cellular debris. Analysis and quantification have been done using the FACSDiva software (BD Biosciences, Franklin Lakes, USA).

### Statistical analysis

Data were expressed as means ± standard error of the mean (SEM) and comparisons between transgenic and control were done using t-test. We used GraphPad Prism version 5.03 (GraphPad software, CA, USA).

## Supporting Information

Figure S1 **Weight modifications in control and hSOD1^G93A^ mice.** Weight changes in control (red) and hSOD1^G93A^ (green) mice were weekly recorded from P56 to the end of the life of the transgenic mice. Statistical analysis: data are expressed as means ± standard error of the mean (SEM), t-test, * p<0.05, ** p<0.01, *** p<0.001.
(TIF)

Figure S2 **FACS analysis of astrocytes in control and hSOD1^G93A^ mice spinal cords at pre-symptomatic age.** The number of spinal astrocytes from hSOD1^G93A^ animals and their control littermates was assessed by flow cytometry using the pan-astrocyte marker Aldh1L1. (A–C) - Representative flow cytometry analysis dot plot astrocyte profiles. A - Negative control (without Aldh1L1 staining). B - control and C - hSOD1^G93A^ spinal astrocytes at P30. In both B - Controls and C hSOD1^G93A^ surrounded areas, designed as “P3", correspond to the labeled cells. The X-axis represents the intensity of fluorescence and the Y-axis the size of the cells.
(TIF)

Figure S3 **FACS analysis of GFAP^+^ astrocytes in control and hSOD1^G93A^ mice spinal cords.** (A–C) - Representative flow cytometry analysis dot plot astrocyte profiles at P30. A - Negative control (without GFAP staining). B - Control and C - hSOD1^G93A^ spinal astrocytes. (E–G) - Representative flow cytometry analysis dot plot astrocyte profiles at P60. E - Negative control (without GFAP staining). F - Control and G - hSOD1^G93A^ spinal astrocytes. (I–K) - Representative flow cytometry analysis dot plot astrocyte profiles at P120. I - Negative control (without GFAP staining). J - Control and K - hSOD1^G93A^ spinal astrocytes. In all profiles, surrounded areas, designed as “P3", correspond to the labeled cells. (D, H, L) - Quantifications of GFAP^+^- astrocytes in control and hSOD1^G93A^ animals at D - pre-symptomatic (P30), H - early-symptomatic (P60) and L - end-stages. The X-axis represents the intensity of fluorescence and the Y-axis the size of the cells. Statistics: t-test; *p<0.05.
(TIF)
