# Supplementary figures and images for: Early Functional Deficit and Microglial Disturbances in a Mouse Model of Amyotrophic Lateral Sclerosis
Source: PLoS One. 2012 Apr 25;7(4):e36000. doi: 10.1371/journal.pone.0036000 (PMC3338492; doi:10.1371/journal.pone.0036000)

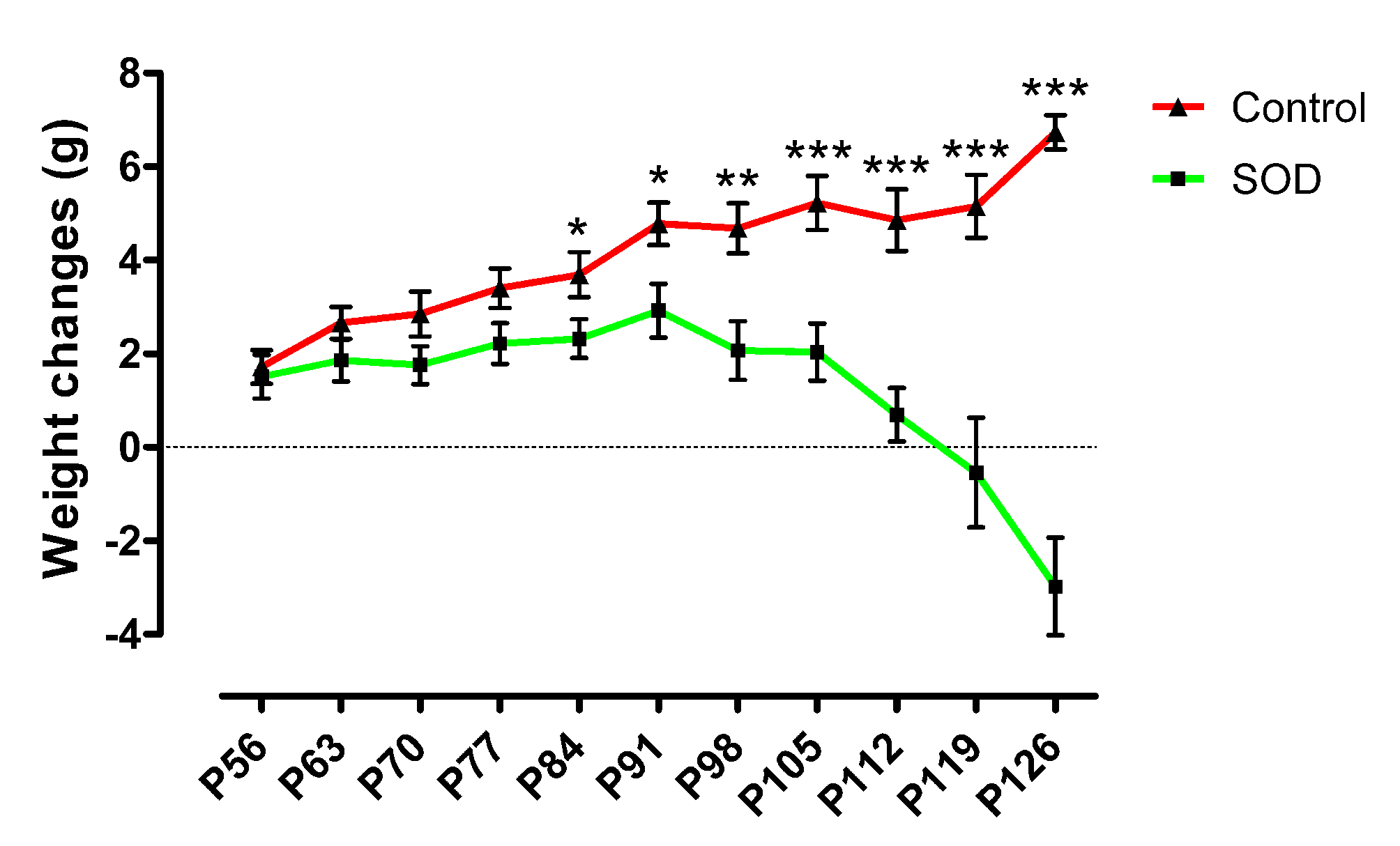

Supplement: Figure S1 — Weight modifications in control and hSOD1G93A mice. Weight changes in control (red) and hSOD1G93A (green) mice were weekly recorded from P56 to the end of the life of the transgenic mice. Statistical analysis: data are expressed as means ± standard error of the mean (SEM), t-test, * p<0.05, ** p<0.01, *** p<0.001. (TIF) [file pone.0036000.s001.tif]

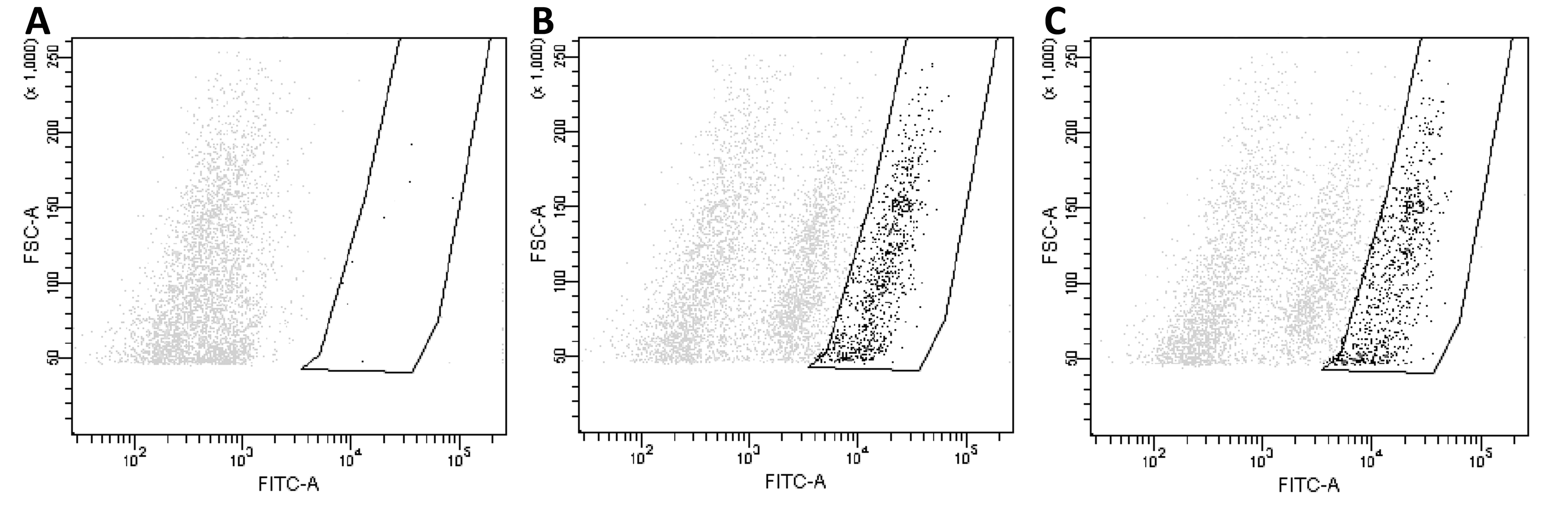

Supplement: Figure S2 — FACS analysis of astrocytes in control and hSOD1G93A mice spinal cords at pre-symptomatic age. The number of spinal astrocytes from hSOD1G93A animals and their control littermates was assessed by flow cytometry using the pan-astrocyte marker Aldh1L1. (A–C) - Representative flow cytometry analysis dot plot astrocyte profiles. A - Negative control (without Aldh1L1 staining). B - control and C - hSOD1G93A spinal astrocytes at P30. In both B - Controls and C hSOD1G93A surrounded areas, designed as “P3", correspond to the labeled cells. The X-axis represents the intensity of fluorescence and the Y-axis the size of the cells. (TIF) [file pone.0036000.s002.tif]

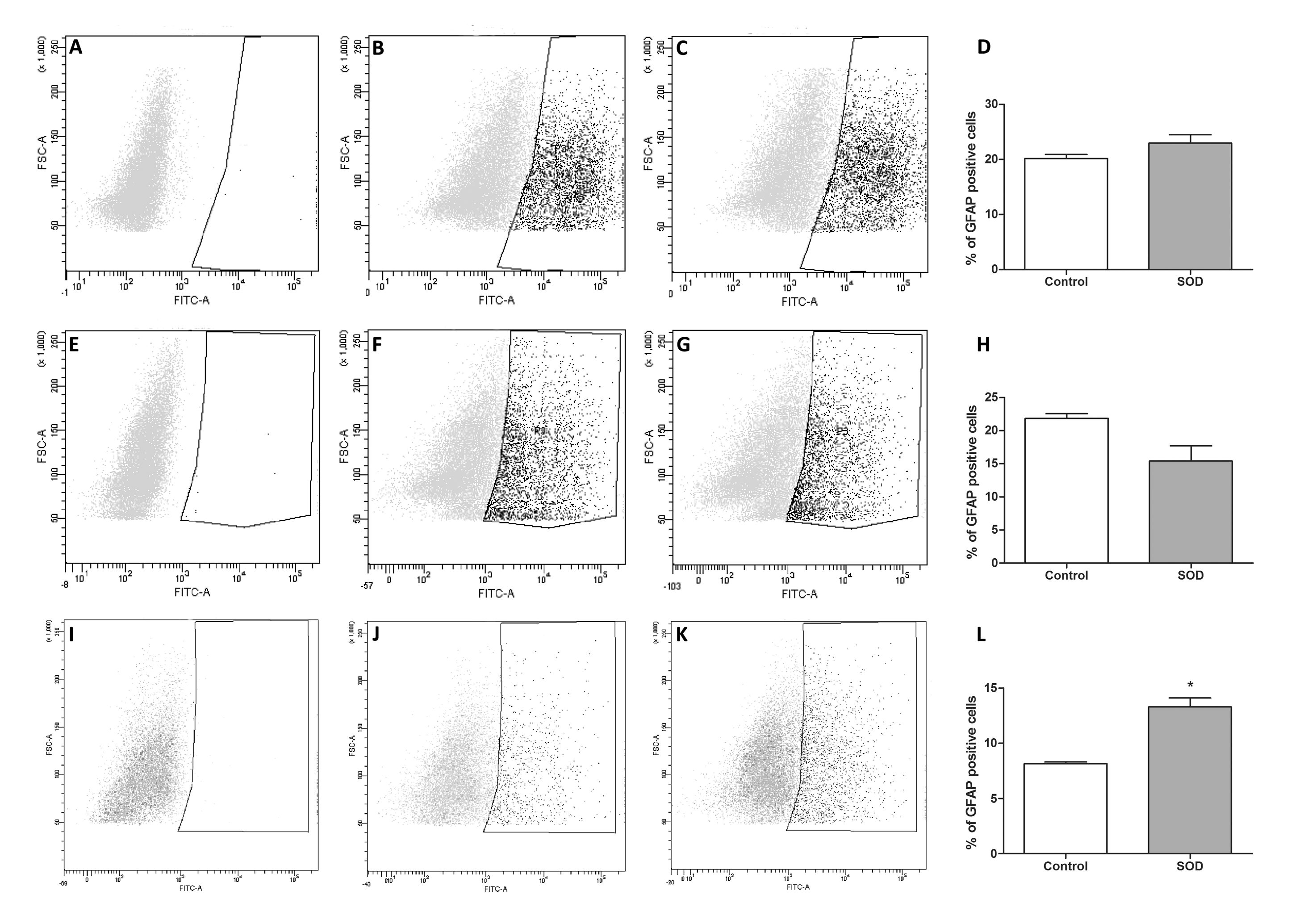

Supplement: Figure S3 — FACS analysis of GFAP+ astrocytes in control and hSOD1G93A mice spinal cords. (A–C) - Representative flow cytometry analysis dot plot astrocyte profiles at P30. A - Negative control (without GFAP staining). B - Control and C - hSOD1G93A spinal astrocytes. (E–G) - Representative flow cytometry analysis dot plot astrocyte profiles at P60. E - Negative control (without GFAP staining). F - Control and G - hSOD1G93A spinal astrocytes. (I–K) - Representative flow cytometry analysis dot plot astrocyte profiles at P120. I - Negative control (without GFAP staining). J - Control and K - hSOD1G93A spinal astrocytes. In all profiles, surrounded areas, designed as “P3", correspond to the labeled cells. (D, H, L) - Quantifications of GFAP+- astrocytes in control and hSOD1G93A animals at D - pre-symptomatic (P30), H - early-symptomatic (P60) and L - end-stages. The X-axis represents the intensity of fluorescence and the Y-axis the size of the cells. Statistics: t-test; *p<0.05. (TIF) [file pone.0036000.s003.tif]
